# Reduced performance of community bednets against pyrethroid-resistant *Anopheles funestus* and *Anopheles gambiae*, major malaria vectors in Cameroon

**DOI:** 10.1186/s13071-022-05335-2

**Published:** 2022-06-26

**Authors:** Emilie S. Ngongang-Yipmo, Magellan Tchouakui, Benjamin D. Menze, Leon M. J. Mugenzi, Flobert Njiokou, Charles S. Wondji

**Affiliations:** 1 Centre for Research in Infectious Diseases (CRID), P.O. Box 13501, Yaoundé, Cameroon; 2 Parasitology and Ecology Laboratory, Department of Animal Biology and Physiology, Faculty of Science, University of Yaoundé 1, P.O. Box 812, Yaoundé, Cameroon; 3 Department of Vector Biology, Liverpool School of Tropical Medicine, Pembroke Place, Liverpool, L35QA UK; 4 Department of Biochemistry and Molecular Biology, Faculty of Science, University of Buea, P.O. Box 63, Buea, Cameroon

**Keywords:** Malaria, LLINs, Insecticide resistance, *Anopheles funestus*, *Anopheles gambiae*, Post-exposure, Longevity, *CYP6P9a*, *GSTe2*

## Abstract

**Background:**

Long-lasting insecticidal nets (LLINs) are a vital tool in the fight against malaria vectors. However, their efficacy in the field can be impacted by several factors, including patterns of usage, net age, mosquito resistance and the delayed mortality effect, all of which could influence malaria transmission. We have investigated the effectiveness of the various brands of LLINs available in markets and households in Cameroon on pyrethroid-resistant mosquitoes and assessed their post-exposure effect.

**Methods:**

Following quality control assessment on a susceptible laboratory mosquito strain, we evaluated the immediate and delayed mortality effects of exposure to LLINs (both newly bough LLINst and used ones collected from households in Elende village, Cameroon, in 2019) using standard WHO cone tests on *Anopheles gambiae* and *Anopheles funestus* populations collected from the Centre region of Cameroon. Alive female mosquitoes were genotyped for various resistance markers at different time points post-exposure to evaluate the impact of insecticide resistance on the efficacy of bednets.

**Results:**

The laboratory-susceptible strain experienced high mortality rates when exposed to all pyrethroid-only brands of purchased nets (Olyset® Net, Super Net, PermaNet® 2.0, Yorkool®, Royal Sentry®) (Mean±SEM: 68.66 ± 8.35% to 93.33 ± 2.90%). However, low mortality was observed among wild *An. funestus* mosquitoes exposed to the bednets (0 ± 0 to 28 ± 6.7%), indicating a reduced performance of these nets against field mosquitoes. Bednets collected from households also showed reduced efficacy on the laboratory strain (mortality: 19–66%), as well as displaying a significant loss of efficacy against the local wild strains (mortality: 0 ± 0% to 4 ± 2.6% for *An. gambiae* sensu lato and 0 ± 0% to 8 ± 3.2% for *An. funestus*). However, compared to the unexposed group, mosquitoes exposed to bednets showed a significantly reduced longevity, indicating that the efficacy of these nets was not completely lost. Mosquitoes with the *CYP6P9a*-RR and L119F-*GSTe2* mutations conferring pyrethroid resistance showed greater longevity after exposure to the Olyset net than their susceptible counterparts, indicating the impact of resistance on bednet efficacy and delayed mortality.

**Conclusion:**

These findings show that although standard bednets drastically lose their efficacy against pyrethroid-resistant field mosquitoes, they still are able to induce delayed mortality in exposed populations. The results of this study also provide evidence of the actual impact of resistance on the quality and efficacy of LLINs in use in the community, with mosquitoes carrying the *CYP6P9a*-RR and L119F-*GSTe2* mutations conferring pyrethroid resistance living longer than their susceptible counterparts. These results highlight the need to use new-generation nets that do not rely solely on pyrethroids.

**Graphical Abstract:**

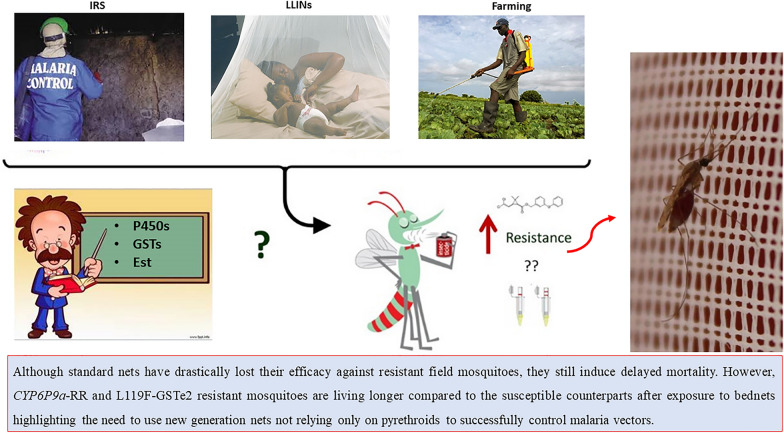

**Supplementary Information:**

The online version contains supplementary material available at 10.1186/s13071-022-05335-2.

## Background

Despite all progress recorded in reducing malaria morbidity and mortality, this disease remains a major public health problem, most notably in sub-Saharan Africa [1]. In Cameroon, malaria is a major public health challenge. The disease was responsible for 25.8% of health facility consultations and 14.3% of deaths in 2018 [2, 3], and hospital morbidity due to malaria was 31.5% among children under 5 years of age and 22.3% among pregnant women. Given the scale of the problem, the Ministry of Public Health and its partners are implementing high-impact interventions to reduce malaria morbidity and mortality. These include: (i) free treatment of uncomplicated and severe malaria for children under 5 years; (ii) seasonal chemoprophylaxis of malaria for children aged 3 to 59 months; and (iii) free distribution of long-lasting insecticidal nets (LLINs) [4] through mass campaigns and intermittent preventive treatment in pregnancy (IPTp) programs [3].

Vector control, particularly through the mass use of LLINs, contributed to a significant decline in the incidence and mortality of the disease between 2000 and 2015 globally and in Cameroon specifically [5]. Inevitably, after many years of prolonged use of pyrethroid insecticides to control agricultural pests and disease vectors, malaria vectors with increasing levels of pyrethroid resistance have emerged, and this has impacted the ability of LLINs to control these mosquito populations [3, 6]. Insecticide resistance has now been reported in malaria vectors against the four main classes of public health insecticides used in the control of these malaria vectors. Different insecticide resistance mechanisms have been reported, including metabolic resistance, target-site mutations, and behavioral changes, as well as cuticular thickening [7, 8].

Many studies in areas of high resistance have revealed a decline in the efficacy of LLINs [9, 10]. Most recently, a study carried out in Papua New Guinea showed that, despite all of the quality-assurance measures, substandard LLINs were distributed during the previous distribution campaign (at least 6 years before the 2013–2019 study), with the good possibility that other countries may have received similar substandard LLINs [11]. Although insecticide-resistant mosquitoes are not killed by immediate contact with insecticides, many studies have found that the effect of exposure can indirectly reduce their disease transmission potential [12, 13]. However, the impact of insecticide resistance on the effectiveness of insecticide-based control interventions and on malaria control is currently a matter of importance in Africa [14–17]. A recent meta-analysis of bioassay studies and experimental hut trials data recently showed that community protection provided by bednets falls rapidly as resistance emerges [18] whereas personal protection is only lost when resistance reaches much higher levels [18]. Also, delayed mortality post-LLIN exposure has been demonstrated in laboratory and field pyrethroid-resistant strains of *An. funestus* (sensu lato [s.l.]) and *An. gambiae* (s.l.) [19, 20]. These studies found that the magnitude of the delayed mortality effects decreases in strains that have developed multiple resistance mechanisms and/or compensatory mutations [19, 21]. One of these studies [21] also showed that mosquitoes carrying the 119F-*GSTe2* pyrethroid resistance mutation live longer than their susceptible counterparts following exposure to the PermaNet® 2.0 LLIN [21]. Given the rapid increase in resistance intensity observed in Africa on a whole and Cameroon in particular [22–24] and the resurgence of malaria [25–27], we sought to evaluate the efficacy and performance of bednets collected from various distribution points in Cameroon, evaluate the residual effect of household bednets previously distributed by the National Malaria Control Program (NMCP) and quantify the presence of any delayed mortality following LLIN exposure in pyrethroid-resistant populations of *Anopheles funestus* and *Anopheles gambiae*. We also evaluated the impact of metabolic resistance on the longevity post-exposure to the Olyset® Net LLIN, taking advantage of recently detected resistance markers for glutathione S-transferases (GSTs) and cytochrome P450 (CYP)-based metabolic resistance in *An. funestus* [28, 29].

## Methods

### Study site

The study was conducted in Yaoundé, the capital city of Cameroon (3°52′12″ N, 11°31′12″ E) and in Elendé, a village very close to Yaoundé) (3°41′57.27″ N, 11°33′28.46″ E). Elendé village is located about 2 km from Yaoundé Nsimalen International Airport and near the Mefou River. The climate is equatorial Guinean, and the vegetation consists of equatorial forest that is increasingly being transformed for agricultural activities and infrastructure. Road-building and deforestation activities are noticeable in this locality. Elendé village is also near streams, swamps and tributaries of major rivers. These permanent water bodies provide favorable conditions for the multiplication of *Anopheles* larvae. People in this village benefited from the bednets distributed during the free LLIN distribution campaign 4 years prior to the present study.

### Collection of new bednets sold commercially in the city of Yaoundé and used in households in Elendé

**Fig. 1:**
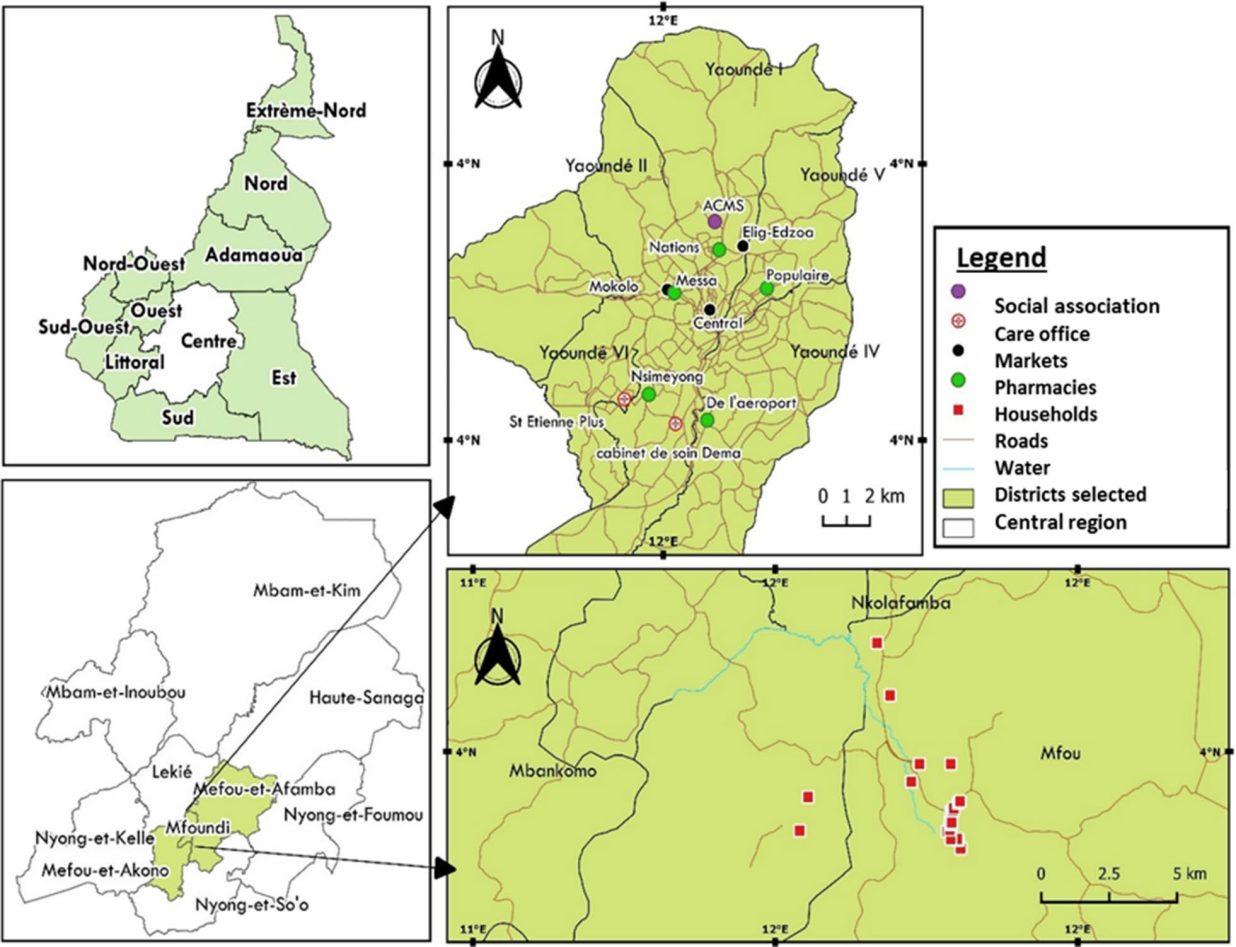
Map of study area, showing the net collection sites in Yaoundé and Elendé

Bednets were purchased from the ACMS (‘Association Camerounaise de Marketing Social’), which is a national distributor of bednets in Cameroon, at three markets, five pharmacies and two health centers in the city of Yaoundé (Fig. 1). Community-based, cross-sectional surveys were carried out in this locality to assess the residual effects of nets distributed by the NMCP during previous campaigns (2015) using a questionnaire. These surveys involved collecting and replacing one net in each of 30 selected households (Fig. 1) based on the principle of exchange with new nets. Interviews were not conducted in houses with no bednet; however, reasons for not having nets in such households were recorded. The questionnaire was adapted from the WHO questionnaire [30]. All nets bought and collected were labeled appropriately and brought back to the Center for Research in Infectious Diseases (CRID) for assessment of their physical integrity and for the WHO cone test.

### Mosquito collection

#### Collection of wild* An. funestus* s.l. females in Elendé

Blood-fed and/or gravid female *An. funestus* were collected indoors between 06:00 a.m and 11:00 a.m in November 2019 using a Prokopack electric hoover (John W Hook, Gainesville, FL, USA) and subsequently kept in a cage covered with a wet towel. The collected samples were then transported to the CRID insectary and separated into the *An. funestus* group and the *An. gambiae* complex using a binocular magnifying glass and the morphological identification key for *Anopheles* mosquitoes [31, 32]. *Anopheles funestus* s.l. mosquitoes were transferred into paper cups, fed with a 10% glucose solution for 4–5 days to become fully gravid before subjected to forced egg-laying. F1 females were used for the bioassays.

#### Larval collection of* An. gambiae* s.l. in Yaoundé

Larval collections were performed in breeding sites around several districts of the city of Yaoundé, namely Ngousso (3°52′48.72″ N, 11°32′24″ E) (in various locations not far from houses), Nkolbisson (3°52′16.32″ N, 11°27′3.679″ E) (near rice crops and also near houses) and Nkolondom (3°57′21″ N, 11°29′53″ E) (near vegetable farms), including from semi-permanent and temporary water bodies at these locations. *Anopheles* larvae of all stages were collected by the dipping method and transported to the CRID insectary for rearing to the adult stage; adult females used for testing. The performance of the bednets was evaluated on these local resistant strains.

### Susceptible strain

The laboratory susceptible strain used in the study is the *Anopheles coluzzii* “Ngousso” strain. This strain originated from the district of Ngousso in Yaoundé and has been colonized since 2006. It is fully susceptible to both permethrin and deltamethrin (mortality rate: ≥ 98% after 1-h exposure to WHO-impregnated papers). Adult mosquitoes of this insecticide-susceptible strain were blood-fed on rabbits and then stimulated to lay eggs. The collected eggs were placed in paper cups containing water for hatching. Once the eggs hatched, the larvae were transferred to rearing tanks and fed with TetraMinBaby® (Tetra Werke, Melle, Germany). The resulting adult females of new generations were used for susceptibility testing to assess the efficacy of the bednets on mosquitoes of this susceptible strain.

Random crosses were performed between FANG and FUMOZ-R, two *An. funestus* laboratory strains, for several generations to bring the *CYP6P9a* allele conferring metabolic resistance to pyrethroids into a susceptible genetic background. FUMOZ-R is a strain that originates from southern Mozambique and which has been selected for pyrethroid (permethrin) resistance [33] and kept in a colony since July 2001. The FANG strain originates from southern Angola and has been kept in a colony since January 2003; this strain is fully susceptible to all major vector control insecticides. Previous studies have shown that the *CYP6P9a* alleles conferring resistance in southern Africa is fixed in the FUMOZ-R strain, whereas it is absent in the FANG strain [29]. To perform the crosses, pupae of each strain were collected and put individually into 15-ml Falcon tubes for individual emergence, following which the males of the resistant strain were mixed in the same cage with the females of the susceptible colony for random mating to generate the first filial generation (F1). Biossays were performed using F_5_ offspring.

### WHO cone bioassays

Cone bioassays were performed according to standard WHO procedures [30]. Five 25 × 25-cm squares were cut from each net, wrapped in aluminum foil and kept at 4 °C until the insecticide activity tests were performed. Each piece was tested with 10 mosquitoes (50 mosquitoes for each LLIN). The experiment was carried out at an ambient temperature of 25 °C ± 2 °C and 80% ± 10% relative humidity. Mosquitoes were exposed to the insecticide nets for 3 min using plastic cones, following which they were gently but rapidly removed from the cones using a mouth aspirator and transferred into paper cups. Knock-down was recorded 60 min after exposure. Mosquitoes were then fed with a 10% sugar solution on soaked cotton balls and mortality was recorded 24 h post-exposure.

### Assessment of the quality and performance of new bednets

All of the new nets bought at the various commerical locations were tested with the susceptible *An. coluzzii* laboratory strain “Ngousso” to assess their quality. Of the better-performing nets (those showing optimal efficacy), a few were selected and tested with the wild *An. funestus* s.l. and *An. gambiae* s.l. mosquitoes to assess the impact of resistance on the efficacy of these nets. Mosquitoes used as ‘controls’ for these tests were those exposed to an untreated net (negative control) and those exposed to a net that has already been approved for efficacy (positive control).

### Assessment of physical integrity and residual efficacy of bednets collected from households

In Elendé, we collected one net in each of 30 randomly chosen households for assessing the physical integrity and the residual efficacy of the nets after many years of use. The physical integrity of the bednets was assessed by checking and counting the holes in the nets using the protocol in the WHO Pesticide Evaluation Scheme (WHOPES) [8]. The total number of holes was categorized by size and position on the net (roof, upper, lower and seams).

The residual efficacy of the nets was assessed using WHO cone tests. All nets were first tested with the susceptible strains and then from the best-performing nets, nine nets were selected and tested on wild *An. funestus* s.l. (F_1_) and *An. gambiae* s.l. (F_0_) using the two categories of control described above.

### Evaluating the effect of the Olyset Net on longevity of the vectors after exposure

F_5_ progeny resulting from the random crosses between the FANG and FUMOZ-R strains were used to evaluate the influence of the recently detected *CYP6P9a* resistance marker on the longevity of mosquitoes following exposure to the LLINs. Also, the impact of the L119F-*GSTe2* resistance marker on the longevity of resistant mosquitoes was investigated using the F_1_ individuals from wild *An. funestus* collected in Elendé. Hybrids and wild *An. funestus* mosquitoes were tested in four replicates using the Olyset Net (Sumitomo Chemical Co., Ltd., Tokyo, Japan) in the cone asssay. After exposure, the mosquitoes that were alieve were monitored daily (control group: *N* = 329 for FUMOZ-R/FANG and *N* = 470 for field *An. funestus* from Elendé; experimental group, *N* = 133 and 270 for FUMOZ-R/FANG strain and field *An. funestus* respectively).

After a 24-h holding period, all of the dead mosquitoes were counted and removed. The mosquitoes still alive were collected and kept in batches of 10 mosquitoes per cup for daily monitoring. Dead mosquitoes were counted each morning and removed from the cups and those still alive were counted and fed with a 10% sucrose solution every day (from the beginning to the end of the experiment).

The *CYP6P9a* and the L119F-*GSTe2* resistance markers were genotyped in dead mosquitoes, and the mortality curves were compared between exposed and unexposed mosquitoes as well as between the genotypes to assess the association with a potential increased ability to survive after exposure to the insecticide-impregnated net.

### Genotyping of resistance markers in *An. funestus* sensu stricto and *An. gambiae* s.l.

In *An. funestus*, the allele-specific PCR (AS-PCR) method was used to genotype the L119F-*GSTe2* marker [34, 35] whereas the *CYP6P9a* marker was genotyped using PCR–RFLP method as previously described [29]. In *An. gambiae* s.l., the L1014F-*kdr* and N1575Y-*kdr* mutations involved in pyrethroid and dichlorodiphenyltrichloroethane (DDT) resistance in *An. gambiae* s.l. were genotyped using the TaqMan assay method as described by Bass et al. [36].

### Data analysis

The mortality rate for each test after a 24-h holding period following the 3 min of exposure to LLINs was calculated in Microsoft Excel 2010 (Microsoft Corp., Redmond, WA, USA). These results were aggregated to determine whether the net meets WHO efficacy requirements with susceptible mosquitoes and were interpreted as follows: (i) for “optimal efficacy,” mortality ≥ 80% or knockdown ≥ 95%; (ii) for “minimal efficacy,” mortality ≥ 50% or knockdown ≥ 75%; (iii) for “non-effective,” mortality < 50% or knockdown < 75%.

Statistical analyses were performed using the online VassarStats and Medcalc software packages. Chi-square (*χ*^2^) and the odds ratio (OR) were used as statistical tests to compare the effect of the resistant marker on delayed mortality by comparing the frequency of resistant allele between mosquitoes at different time points. GraphPad Prism 7.00 (GraphPad Software Inc., San Diego, CA, USA) was used to construct graphs. R version 3.3.1 for Windows 10 was used to construct and compare the mortality curves between the exposed and unexposed groups and between genotypes for each marker.

## Results

### Nets collected

In total, 45 nets (Additional file 1: Table S1) of five different brands were bought from various commerical outlets in Yaoundé; these brands were Olyset Net (containing 2% permethrin), Super Net (Sankari Plastic, Karur, Tamil Nadu, India; containing 2% permethrin), Perma® Net 2.0 (Vestergaard, Lausanne, Switzerland; containing 1.4–1.8 g/kg ± 25% deltamethrin), Yorkool® (Tianjin Yorkool International Trading Co., Ltd, Tianjin, China; containing 55 mg/m^2^ deltametrin) and Royal Sentry® (Clariant International AG, Muttenz, Switzerland; containing 261 mg/m^2^ alpha cypermethrin ± 25%). Super Net was the most abundant (34%). In Elendé, 30 nets (Additional file 1: Table S2) (5 brands) were collected from 30 households (1 net from each household). In addition to the five LLINs mentioned above, two other LLINs were identified by their labels: PandaNet® (LIFE IDEAS Biological Technology Ltd., Jiangmen city, China; containing 1.8 g/kg ± 25% deltamethrin) and Interceptor® (BASF, Ludwigshafen, Germany; containing alpha cypermethrin 200 mg/m^2^). The Olyset net was the most recurrent (63%). Two of the collected nets were without labels, making identification of the brand impossible.

### Mosquitoes collected in Yaoundé and Elendé

Approximately 900 blood-fed wild *An. funestus* s.l. females (F_0_) were collected in Elendé. From this collection, 1800 F_1_ females were used to evaluate the performance of the nets. Around 400 *An. gambiae* s.l. females (F_0_) were collected in Yaoundé neighborhoods (for all three quarters: larvae collection). After rearing, 2750 and 1750 *An. coluzzii* “Ngousso” (susceptible laboratory strain) females were used to evaluate the efficacy of new nets and nets collected from households in Elendé, respectively.

### Species identification

Molecular identification of 90 *An. funestus* s.l. females from Elendé revealed the presence of only one species of this group, namely *An. funestus* sensu stricto (s.s.) A total of 30 *An. gambiae* were analyzed for species identification. The results revealed that the samples of *An. gambiae* s.l. collected in Yaoundé consisted of two species: *An. coluzzii* and *An. gambiae* s.s., with* An. coluzzii* the majority species (18/30 individuals).

### Effect of purchased new nets from Yaoundé outlets in tests with the susceptible strain

**Fig. 2:**
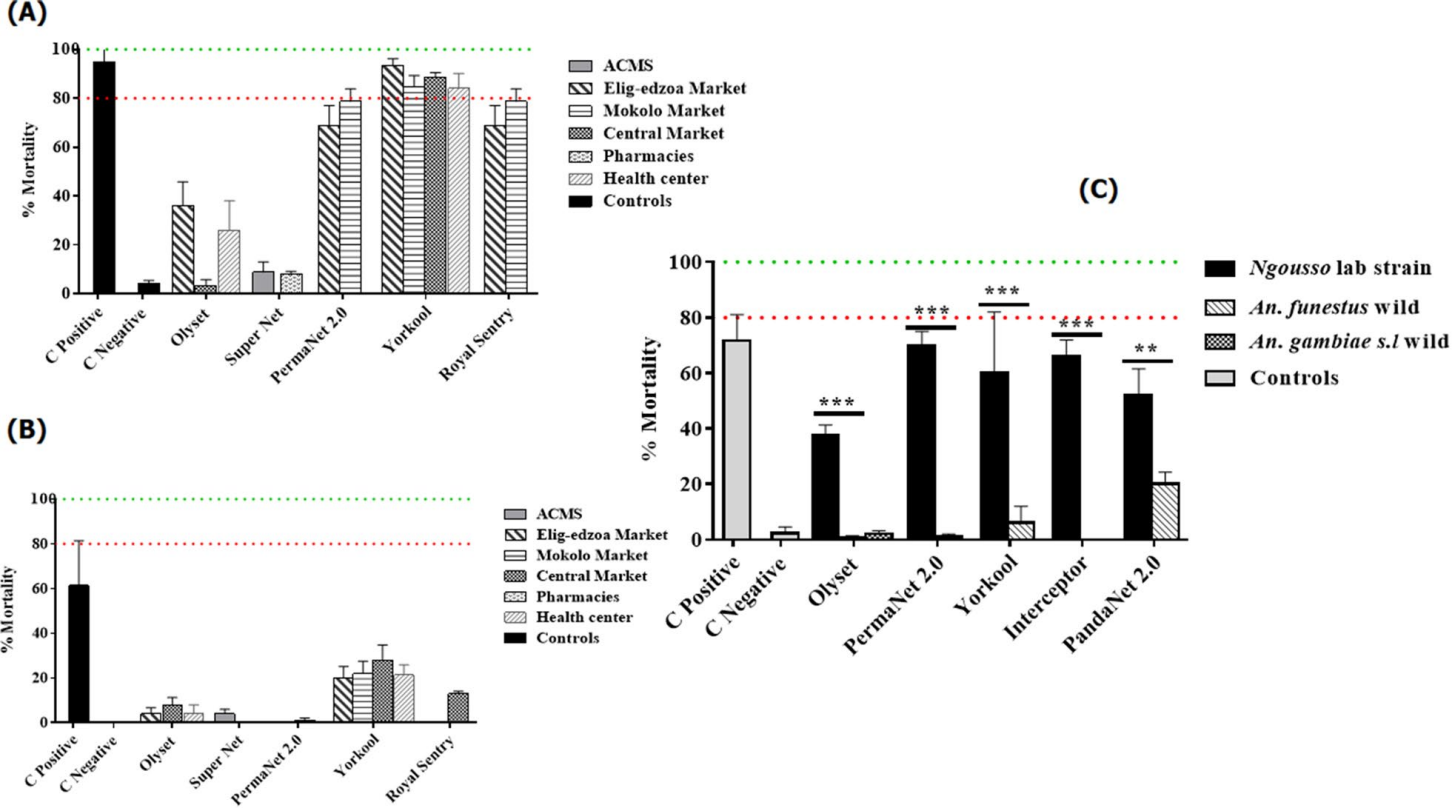
Efficacy and performance of new nets and nets collected from households in Elendé. **a** Mortality rate of susceptible *Anopheles coluzzii* laboratory strain “Ngousso” mosquitoes tested with new LLINs. **b** Mortality rate of wild *Anopheles funestus* s.l. mosquitoes tested with new LLINs. **c** Mortality rate (Mean± SEM) of susceptible wild strains of *An. funestus* s.l. and *Anopheles gambiae* s.l. tested with LLINs collected from households in Elendé. Abbreviations: ACMS, ‘Association Camerounaise de Marketing Social’; C control; LLINs, long-lasting insecticidal nets; * indicates the level of significance in the difference of mortality rate between the field resistant strains compared to suceptible lab strain

A high mortality rate was obtained in tests with the positive control net (Mean± SEM: 95% ± 5%) (a net whose efficacy had already been approved). However, the mortality rate was very low in tests with the Olyset (*χ*^2^ = 70.1; *P * < 0.0001) and Super Net LLINs (*χ*^2^ = 60.6; *P *< 0.0001), all of which were impregnated with permethrin, with mortality rates ranging from 3.30% ± 2.40 to 36% ± 9.79% for all of the bednets. The mortality rate in the susceptible strain was higher in tests with the other brands of bednets (PermaNet 2.0, Yorkool, Royal Sentry), with mortalities ranging from 68.66% ± 8.35% to 93.33% ± 2.90% (Fig. 2a). No significant difference was observed in the mortality rate of the positive control compared to the PermaNet 2.0 (*χ*^2^ = 1.3; *P* = 0.2), Yorkool (*χ*^2^ = 0.9; *P* = 0.31) and Royal Sentry (*χ*^2^ = 1.3; *P* = 0.2) LLINs.

Based on the mortality rates obtained with the susceptible strain, 46.6% (21/45) of nets were classified as non-effective, 17.77% (8/45) presented minimal efficacy and 35.55% (16/45) displayed optimal efficacy. All nets with optimal efficacy were of the brands PermaNet 2.0, Yorkool and Royal Sentry, mostly manufactured in 2018, and all those classified as non-effective were of the brands Olyset Net and Super Net, manufactured in 2014 and 2016.

### Performance of new nets in tests with wild mosquitoes

A total of 1300 F_1_ females of *An. funestus* s.l. and 200 wild *An. gambiae* s.l. were used in tests involving the nets with optimal efficacy. A decrease in performance was observed for all nets, including the positive control, with low mortality rates observed on the wild strain of *An. funestus* s.l. (mortality ranging from 0% ± 0% to 28% ± 6.79%) (Fig. 2b). A significant difference in mortality rates was obtained with the two positive controls, with the “Ngousso” strain showing a mortality rate of 87.6% and the wild populations of *An. funestus* s.l. showing a mortality rate of 61.3% (*χ*^2^ = 9; *P* = 0.003). The same result was obtained with the PermaNet 2.0 LLIN (mortality ranging from 78.6% with the susceptible strain to 1% with the wild strain) (*χ*^2^ = 62.2; *P * < 0.0001), the Yorkool LLIN (mortality ranging from 93.33% to 22%) (*χ*^2^ = 38.9; *P *< 0.0001) and the Royal Sentry LLIN (mortality ranging from 78.6% to 13%) (*χ*^2^ = 42.9; *P * < 0.0001). Although the Olyset and Super Net LLINs did not show greater efficacy in test with the susceptible strain, a significant decrease in mortality rate in the wild strain compared to the susceptible strain was observed. The mortality rate with the Olyset bednet ranged from 36% in the susceptible strain to 8% in the resistant strain (*χ*^2^ = 11.31; *P* = 0.0008), and with the Super Net bednet, from 8.2% in the susceptible strain to 0% in the resistant strain (*χ*^2^ = 4.1; *P* = 0.04) (Fig. 2a, b).

These results show that insecticide resistance reduces the performance of LLINs. Due to the low numbers of *An. gambiae* s.l. obtained, only the performance of the Super Net LLINs from ACMS was tested. Very low mortality rates were observed for these nets, ranging from 0% ± 0% to 10% ± 6.14%.

### Physical integrity of bednets

**Fig. 3:**
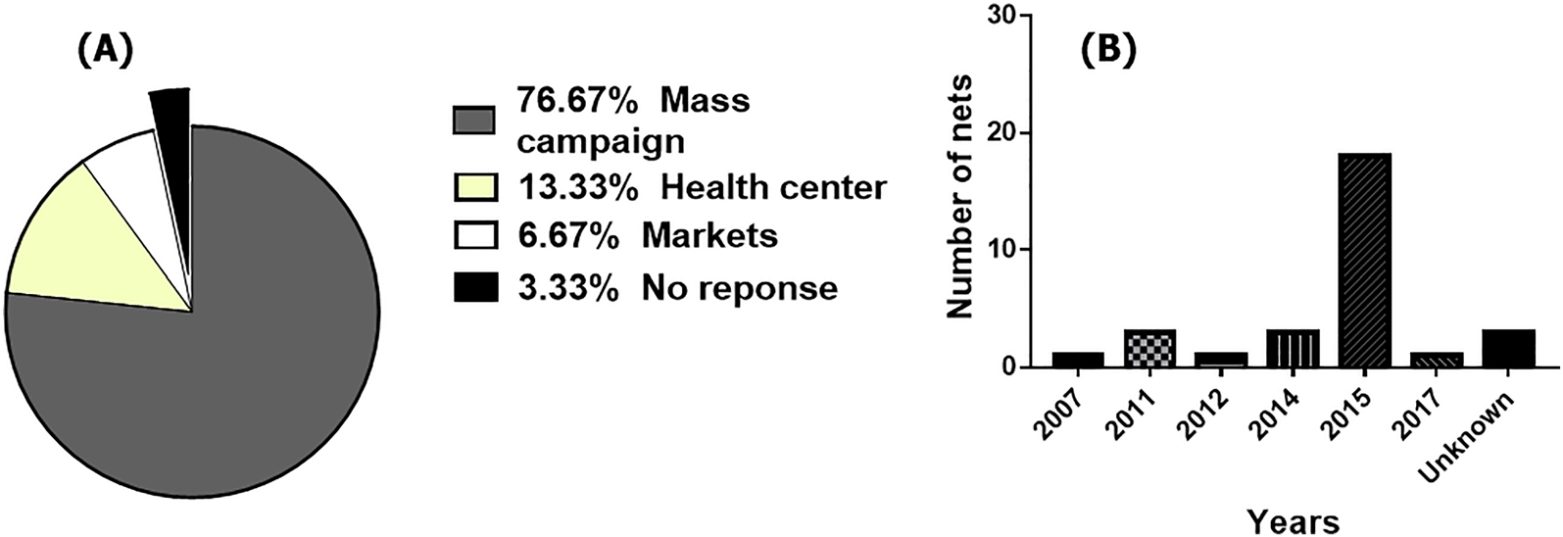
Information on nets collected from households. **a** Origin of LLINs, **b** age of LLINs

Of the respondents to the 30 household questionnaires, 56.67% were heads of the household, the majority of whom (53%) had secondary education. Of the households surveyed, 86.66% had at least one child, and about 56.66% had a bednet in thehome. In addition, 6.66% of respondents mentioned not sleeping under a net mainly because of the age of their nets or simply becuse of not having problems with insect nuisance. The Olyset brand, largely (76.67%) from the 2015 free distribution campaign organized by the Ministry of Public Health, was widely represented (63.33%) (Fig. 3a). However, the 01 bednet with a label from the Republic of Nigeria was also found in this village(3.33%). In addition, very old LLINs (dating back several years, 2007–2015) were collected (Fig. 3b). Of the total number of respondents to the questionnaire, 60% admitted to having washed their LLINs at least once with local soap (44.44%) or detergent powder (16.67%). Mortality rates were assessed for each category (washed and unwashed nets). The mortality rate was slightly higher with washed nets (49% ± 4.84%) compared to unwashed nets (38.16% ± 5.88%). However, this difference was not significant (*χ*^2^ = 0.33; *P* = 0.56). Holes caused by children were the most common type of damage. The physical condition of each LLIN collected was recorded according to the size of holes, and size 1 holes were found to be the most prevalent on all sides of the nets. The proportional hole index [37] allowed each LLIN to be categorized as: either “good,” acceptable” or “torn.” Accordingly, 30% of the LLINs were classified as good, 40% as acceptable and 30% as torn and requiring replacement as soon as possible. However, the percentage of nets still usable in the village (good + acceptable) was 70%.

### Residual efficacy of nets collected in Elendé

A total of 500 wild *An. funestus* s.l. females and 200 wild *An. gambiae* s.l. females were used to assess the performance of nets collected from households (which had been used for at least 3 years). Figure 2C shows the mortality rates obtained with the different strains of *Anopheles*. For all nets, the mortality rate was < 80% with the susceptible “Ngousso” strain. The mortality rate was lower following exposure to the Olyset LLIN (37.6 ± 3.68%) and relatively higher following exposure to the other brands of LLINs (PermaNet 2.0, Yorkool, Interceptor, PandaNet 2.0), with the mortality rate ranging from 52% ± 9.52 to 70% ± 5.1%. However, a significant difference was observed between the mortality rate obtained with the susceptible “Ngousso” strain of mosquitoes and that obtained with the wild strain of *An. funestus* s.l. exposed to the Olyset net (mortality rate ranged from 37.6 to 0.66%) (χ^2^ = 21.3; *P * < 0.0001). The same result was obtained with the PermaNet 2.0 (mortality rate ranged from 70% to 1.0%) (*χ*^2^ = 51.5; *P* < 0.0001), Yorkool (mortality rate ranged from 60% to 6%) (*χ*^2^ = 32.6; *P * < 0.0001), Interceptor (mortality rate ranged from 66% to 0%) (*χ*^2^ = 48.8; *P* < 0.0001) and PandaNet 2.0 (mortality rate ranged from 52% to 20%) (*χ*^2^ = 11; *P * < 0.009) LLINs. Figure 2C also shows a very low mortality rate with wild *An.gambiae* s.l. mosquitoes, with mortality ranging from 0% ± 0% to 4% ± 2.66% with Olyset brand nets.

These results show that the LLINs already in use were still effective against the susceptible strain but were ineffective against the field strains. In summary, of the nets collected from households in Elendé, 17/30 (57%) were classified as non-effective when tested against the susceptible strain, 11/30 (37%) displayed minimal efficacy and only 2/30 (6%) were found to be optimally effective.

### Distribution of resistance markers in field *An. funestus* s.s. and *An. gambiae* s.l

The distribution of genotypes for the L119F-*GSTe2* resistance markers in 30 F_1_ *An. funestus* s.s. mosquitoes from Elendé was 13 (43%) 119F/F-RR homozygous resistant, 12 (40%) 119L/F-RS heterozygous and five (17%) L/L119-SS homozygous susceptible. Genotyping of the L1014F-*kdr*-West locus in *An. gambiae* s.l. from Yaoundé revealed a very high frequency of this mutation, with 25 (83%) mosquitoes homozygous resistant, five (17%) heterozygous and 0 (0%) homozygous susceptible. In contrast, the N1575Y mutation was completely absent in this population, which only had the SS genotype detected.

### Delayed mortality post-exposure to Olyset net

**Fig. 4:**
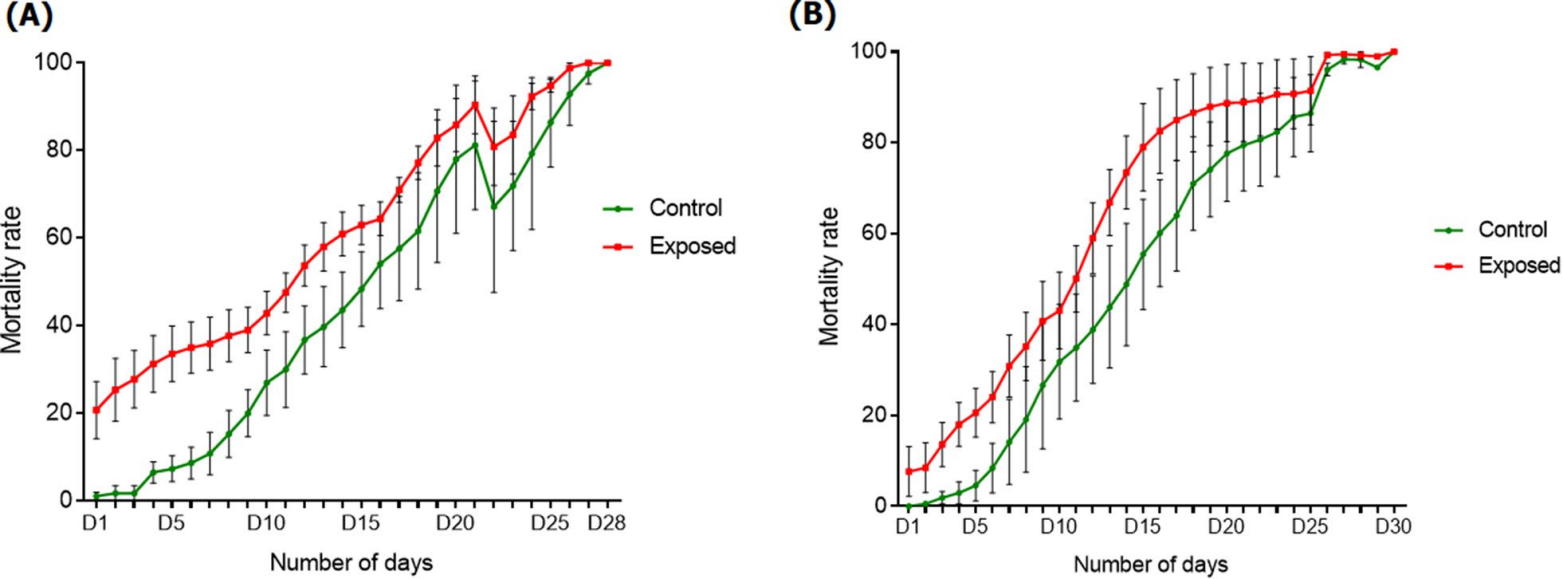
Delayed mortality post-exposure to the treated Olyset bednet. **a** Mortality rate of the* An. funestus* laboratory strain FUMOZ/FANG after exposure to the Olyset bednet. **b** Mortality rate of the *An. funestus* field strain from Elendé after exposure to the Olyset bednet

Comparison of the mortality curve of surviving FUMOZ-R/FANG hybrids following exposure to the Olyset net with that of their unexposed counterparts revealed a higher mortality rate in the exposed group (*χ*^2^ = 25.5 *P *< 0.0001; *χ*^2^ = 10.2 *P* = 0.0014; *χ*^2^ = 3.7, *P* = 0.05) (Fig. 4a). For the *An. funestus* field strain, the same pattern was observed between both groups and was more pronounced from day 1 to day 5 (*χ*^2^ = 49.82 *P *< 0.0001), highlighting delayed mortality after exposure to the Olyset net (Fig. 4b).

### Effect of* CYP6P9a* and L119F-*GSTe2* resistance markerz on the long-term efficacy of the Olyset net

**Fig. 5:**
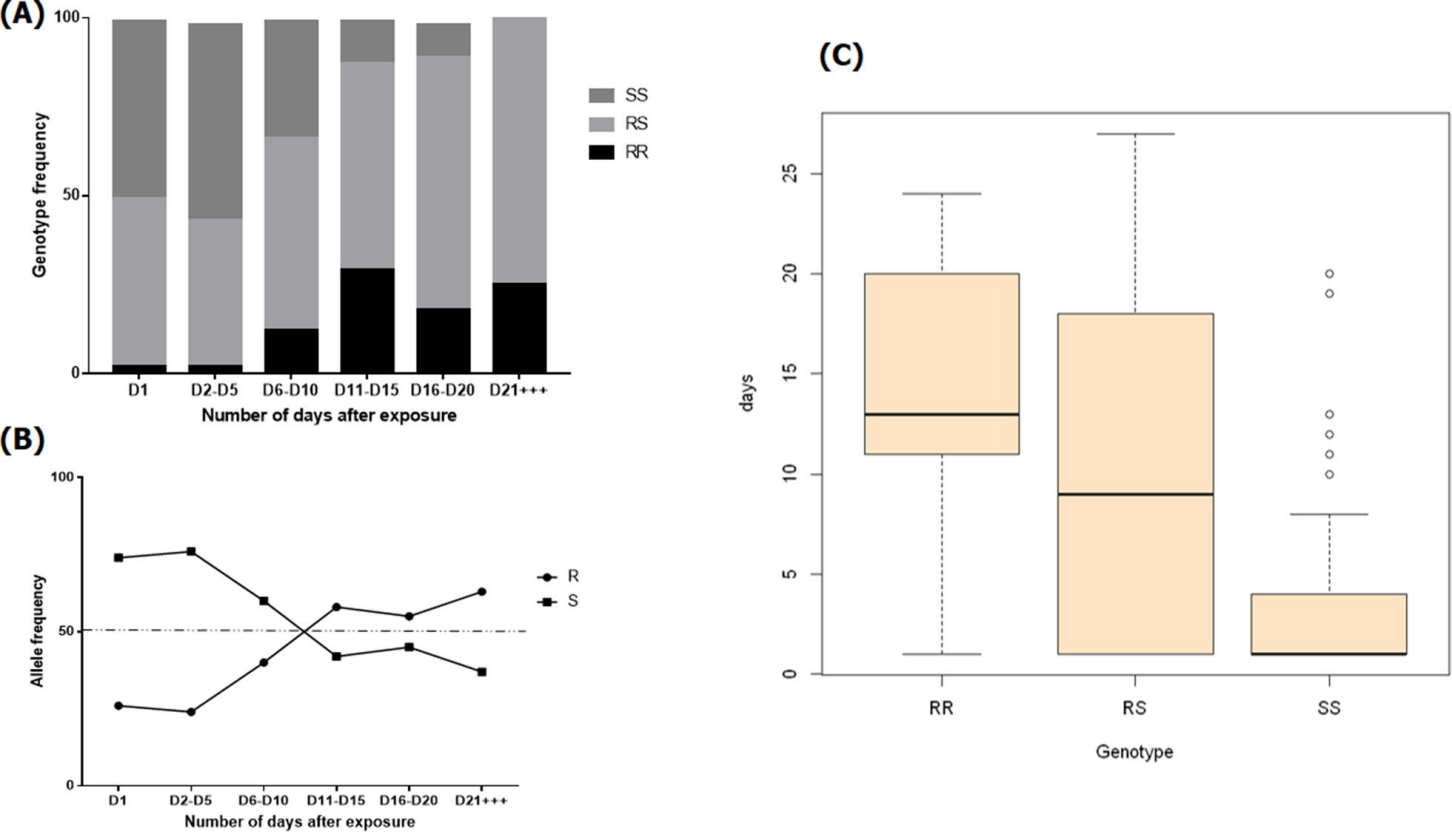
Influence of the *CYP6P9a* mutation on the adult longevity of the *An. funestus* laboratory strain Fumoz × Fang. **a** Distribution of genotypes per interval after exposure, **b** distribution of alleles in surviving mosquitoes at different times post-exposure to the Olyset net, **c** mean number of survival days of mosquitoes with the* CYP6P9a*-RR,* CYP6P9a*-RS and* CYP6P9*a-SS genotypes, respectively, after exposure to the Olyset net

A total of 229 FUMOZ-R/FANG mosquitoes from the group exposed to the Olyset bednet were genotyped for *CYP6P9a* between day 1 (D1) and day 27 (D27) post-exposure to compare the life span between mosquitoes with different genotypes for this marker. In the mosquitoes which were alive after exposure, heterozygous* CYP6P9a*-RS individuals represented 55% of the population whereas 33.63% were homozygous susceptible* CYP6P9a*-SS and 11.36% were resistant homozygous* CYP6P9a*-RR. The frequency of* CYP6P9a*-RR and* CYP6P9a*-RS mosquitoes among the dead mosquitoes analyzed increased significantly from D1 1 to D27 post-exposure, with 2.4% being* CYP6P9a*-RR on D1 versus 25% on D27 (*χ*^2^ = 41.1; *P *< 0.0001), and 47.56% being* CYP6P9a*-RS on D1 versus 75% on D27 (*χ*^2^ = 32.86; *P *< 0.0001); in contrast, the frequency of* CYP6P9a*–SS individuals decreased over this same time period (50% vs. 0%) (*χ*^2^ = 11.7; *P* = 0.0006) (Fig. 5a). This dynamic in the distribution of the resistance marker after exposure to the Olyset net was most apparent in the distribution of alleles from D1 to D27 (Fig. 5b). Mosquitoes with the susceptible allele were the most affected after exposure to the Olyset net as the proportion of living mosquitoes with this allele decreased over time while the proportion of those with the resistant allele increased (Fig. 5b). The mean survival time of* CYP6P9a*-RR mosquitoes after exposure was 14.56 days, of* CYP6P9a*-RS mosquitoes, 10.04 days, and of* CYP6P9a*-SS individuals, 3.39 days; these results confirm that after exposure to the Olyset net, mosquitoes with* CYP6P9a*-SS allele die quicker compared with those with the other genotypes (Fig. 5c). This conclusion was further supported by the OR estimation (Table 1) where* CYP6P9a*-RR mosquitoes displayed a significantly greater longevity compared to their* CYP6P9a*–RS and* CYP6P9a*–SS counterparts (RR vs. RS: OR = 6.8 95%, confidence interval [CI]: 1.2–1.7, *P* = 0.008); RR vs. SS: OR = 687.5, 95% CI 7.41–362, *P* < 0.0001). Similarly, heterozygous mosquitoes also showed significantly higher longevity compared to –SS (OR = 100.6, 95% CI 5.1–253; *P*<0.0001). Therefore, at the allelic level, mosquitoes bearing the* CYP6P9a*-R allele displayed greater ability to survive compared to those with the* CYP6P9a*-S susceptible allele (OR = 5.1, 95% CI: 1.6–2.9; *P* < 0.0001), indicating that possessing the* CYP6P9a*-R resistant allele increases the chance of mosquitoes surviving several days post-exposure to the Olyset net.

**Table 1:** Evaluation of the association between different genotypes of the *CYP6P9a* mutation and female longevity in exposed mosquitoes

Genotypes	(D2-D5) × ( D6-D10)			(D2-D5) × (D11-D15)			(D2-D5) × (D16-D20)			(D2-D5) × (D21+++)		
	OR	95% CI	*P*	OR	95% CI	*P*	OR	95% CI	*P*	OR	95% CI	*P*
RR vs. SS	10	1.62–3.22	0.002*	43.54	2.97–8.5	< 0.0001*	55	3.43–11.93	< 0.0001*	687.5	7.41–362	< 0.0001*
RR vs. RS	4.55	1.14–1.98	0.07	6.71	1.24–1.91	0.01*	5.19	1.15–1.7	0.03*	6.83	1.2–1.7	0.008*
RS vs. SS	2.19	1.09–2.09	0.008*	6.48	1.90–5.60	< 0.0001*	10.58	2.42–8.39	< 0.0001*	100.60	5.16–253	< 0.0001*
R vs. S	2.02	1.06–1.82	0.02*	4.19	1.48–2.59	< 0.0001*	3.71	1.40–2.43	< 0.0001*	5.1	1.62–2.93	< 0.0001*

*CI* Confidence interval, *D* day (post-exposure), *CYP6P9a–RR* homozygous resistant, *CYP6P9a–RS* heterozygous resistant; *CYP6P9a–SS* homozygous susceptible,* OR* odds ratio

*Indicates significant difference between genotypes for the OR

**Fig. 6:**
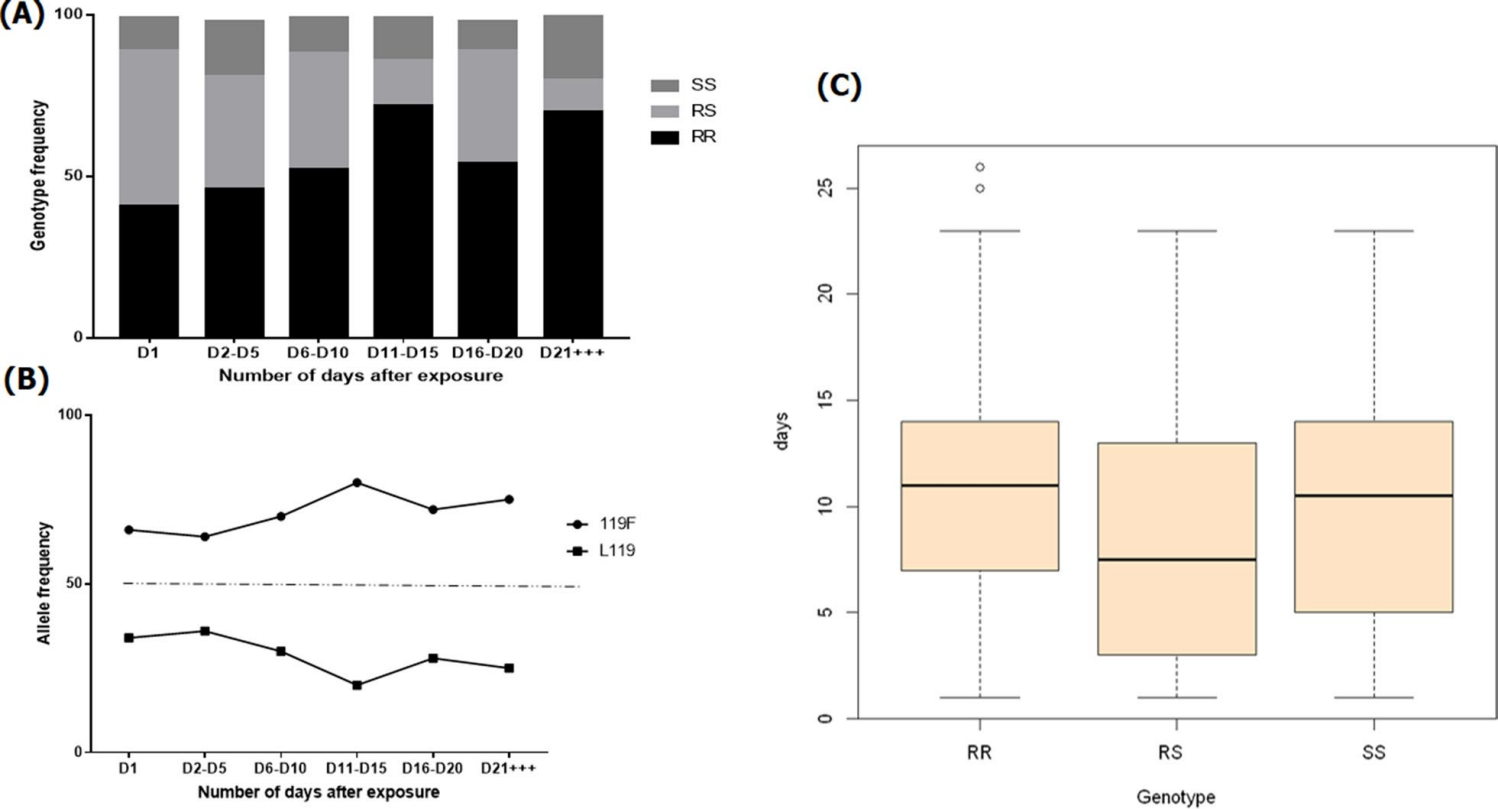
Influence of the L119F-*GSTe2* mutation on the adult longevity of *An. funestus* field strain from Elendé. **a** Distribution of genotypes per interval after exposure, **b** distribution of alleles after exposure in mosquitoes exposed to the net at different time points post-survival in the surviving mosquitoes, **c** Mean number of survival days in mosquitoes bearing the 119F-RR, L119F-RS and L119-SS genotypes, respectively, after exposure to the Olyset net

For the L119F-*GSTe2* resistant marker, genotyping of 351 dead mosquitoes from D1 to D26 post-exposure revealed a predominance of mosquitoes with the L119F-RR genotype (58.40%), followed in decreasing order of prevalence by those with the L119F-RS genotype (28.49%) and those with the L119-SS genotype (13.10%) (Fig. 6a). For this marker, (Table 2), homozygote resistant mosquitoes (L119F-RR) displayed a significantly greater longevity compared to –RS and –SS mosquitoes between D1 and D15 (RR vs RS: OR = 4.75, 95% CI: 1.46–2.30 *P *< 0.0001); RR vs. SS: OR = 3.04, 95% CI: 1.10–2.72; *P* = 0.004). However, between the –RS and –SS mosquitoes, no survival advantage was observed between D1 and D15 (RR vs SS: OR = 0.64, 95% CI: 0.39–1.37; *P* = 0.34). Similarly, for the same time interval, individuals possessing the resistant allele had a greater ability to survive compared to individuals with the susceptible allele (R vs. S: OR = 2.77, 95% CI: 1.19–2.58; *P* = 0.001), indicating that possessing the resistant allele increases the chance of mosquitoes to survive several days post-exposure to the net, similar to the observation made for the* CYP6P9a* marker (Fig. 6b). Because of low number of mosquitoes surviving until D26, no significant difference in mortality between –SS and –RR mosquitoes was observed between D2 and D26 (OR = 1.92, 95% CI: 0.94–1.89; *P* = 0.07); this was also true for –RS (RR vs. RS: OR = 6.47, CI at 95%: 1.69–5.27; *P *< 0.0001) and –SS (RS vs. SS: OR = 3.36, 95% CI: 1.18–4.22; *P* = 0.008) mosquitoes (Table 2). The mean survival time of L119F-RR mosquitoes after exposure was 10.6 days, of L119F-RS mosquitoes, 8.5 days, and of L119F-SS mosquitoes, 9.86 days; these results show that –RR mosquitoes have a higher ability of surviving compared to –RS and –SS mosquitoes (Fig. 6c).

**Table 2:** Evaluation of the association between different genotypes of L119F-*GSTe2* and the longevity of exposed mosquitoes

Genotypes	(D2–D5) × (D6–D10)			(D2–D5) × (D11–D15)			(D2–D5) × (D16–D20)			(D2–D5) × (D21+++)		
	OR	95% CI	*P*	OR	95% CI	*P*	OR	95% CI	*P*	OR	95% CI	*P*
RR vs. SS	2.6	1.01–2.83	0.02*	3.04	1.10–2.72	0.004*	3.3	1.11–3.52	0.006*	1.92	0.94–1.89	0.07
RR vs. RS	1.33	0.85–2.52	0.35	4.75	1.46–2.30	< .0001*	1.42	0.88–1.58	0.25	6.47	1.69–5.27	< .0001*
RS vs. SS	1.94	0.86–2.52	0.12	0.64	0.39–1.37	0.34	0.43	0.32–1.08	0.06	3.36	1.18–4.22	0.008*
R vs. S	1.62	0.93–1.75	0.10	2.77	1.19–2.58	0.001*	1.78	0.97–1.87	0.05*	1.48	0.91–1.61	0.16

*119F-RR* Homozygous resistant, *L119F-RS* heterozygous resistant, *L119-SS* Homozygous susceptible

*Indicates significant difference between genotypes for the OR

## Discussion

Our evaluation of the quality of purchased and used LLINs found that these nets were effective against laboratory mosquitoes and did not perform well against field mosquitoes. However, it is necessary to evaluate the effect of exposure to the most used LLIN, namely the Olyset net, over a long period in order to develop better control strategies.

Based on the nets bought in the city of Yaoundé, the most widely sold nets were the Olyset and Super Nets. The predominance of these two brands of nets has been reported in previous studies conducted in the South-West and North-West regions of Cameroon [38]. Some LLINs retained their effectiveness against susceptible vectors. The low mortality rates obtained with new Olyset and Super nets purchased in markets, pharmacies, and even from the national distributor represents a real problem for vector control. Outside of mass campaigns, ACMS is the main entity in Cameroon that is in charge of distributing LLINs to the population and to certain health services, such as pharmacies. The poor performance and even low effectiveness of these nets indicate that most of these nets are not suitable for vector control and that there is an urgent need to improve the quality of these nets. A similar result was obtained in Papua New Guinea where the authors of the study found that, despite the quality-assurance measures in place, substandard LLINs had been distributed in the country for at least 6 years (2013–2019) [11].

Our evaluation of the performance of these nets on field mosquitoes showed little or no mortality compared to the mortality rate of the susceptible strain. One of the main reasons put forward for this decline in net performance is the development of insecticide resistance in the major malaria vectors, which significantly reduces the effectiveness of nets, as reported in previous studies in Cameroon and other African countries [39–41]. These results show that alternative measures are necessary to control these vectors, such as second-generation nets, which have been shown to be of better efficacy [42–44]. However, the presence and use of nets that are > 3 years old in health institutions or the selling of such aged nets in markets or other commercial setting is a brake on malaria control, as is the use of counterfeit/substandard nets that possibly have ineffective insecticide content and therefore reduced effectiveness. It is in the interest of manufacturers, donors, recipient countries and, most importantly, those relying on LLINs for protection against malaria that the quality-assurance framework for these important commodities remains rigorous, adaptive and transparent.

This study also showed that the Olyset net was the most widely used LLIN in Elendé, followed by the PermaNet 2.0 net. The main source of LLINs in this locality is via free distribution campaigns [38]. About 90% of respondents claimed to use LLINs to protect themselves, regardless of the season, which indicates good coverage. However, all the LLINs collected for evaluation in this locality had holes of varying sizes, mainly size 1. Most of these holes were found on the lower part of the nets (the part that is usually threaded under the mattress). This is not surprising, as this part of the net is the one most handled by persons sleeping in the beds. Several factors influence the physical deterioration of LLINs, such as the environment of the house (wall materials, type of bed and construction), but also socio-economic status and, most importantly, the way the nets are maintained (general handling, washing and repair) [45]. A low rate of repaired LLINs (30%) was recorded. In Gambia, this low rate of repair was attributed to the poor quality of the net material or the location of holes in places that are likely to tear after repair [46]. However, there is no doubt that many nets may have been poorly sewn and still ended up in circulation, increasing the percentage of deteriorated nets in households. It is therefore crucial to establish a strong quality control system for nets being used on the continent.

Of the respondents to the questionnaire, 60% admitted to having washed their LLINs at least once with local soap (44.44%). This observation was in contrast with the observations made in Limbé, Tiko and Buéa where the populations reported using detergent for the most part [38]. The mortality rate obtained with washed LLINs was higher than that with unwashed LLINs (49% vs. 38.16%,) but the difference was not significant. This lack of significance is mainly due to the fact that most of the insecticide is in the net shell, which serves as a reservoir, with only a small portion remaining on the surface. When the net is washed, the insecticide on the surface of the net is washed off with the deposits. New insecticide molecules from the reservoir then migrate to the surface to re-treat the net [30]. This process is repeated until the reservoir is empty. The nets in the Elendé households were mainly covered with particles (dust, smoke mist, grease) that had been deposited on the net fibres, impeding the diffusion of the insecticide. Washing would is therefore essential for greater effectiveness. Several previous studies have shown that when LLINs are hung in houses, dust or fumes can coat the insecticide molecules, inhibiting contact with the mosquitoes and reducing the effectiveness of the nets [47, 48]. Our categorization of LLINs estimated that 30% of the nets collected in Elendé were in good condition, 40% were in acceptable condition and 30% were too torn to be effective; thus, 70% of the nets were still usable. These results indicate that 3 years after the distribution of LLINs, it is urgent that households replace them, which is in line with WHO recommendations. The village of Elendé has not received a net since the last campaign because the LLIN distribution system coordinated by the Ministry of Health and the NMCP is not systematic. In some areas, for example, households are still using nets from the previous campaign (2015) and have not received any nets from the recent 2019 bednet distribution campaign.

Our study showed a residual effectiveness of nets collected from households and also the actual efficacy of LLINs against the susceptible strain *An. coluzzii* “Ngousso,” revealing that only 6% of the LLINs purchased had an optimal efficacy. Also, a decreased effectiveness of the selected LLINs against a field strain was observed, confirming the very high level of pyrethroid resistance in wild mosquitoes [49]. Several studies have demonstrated the inability of previously used and perforated nets to kill field mosquitoes due to pyrethroid resistance in local vectors [4]. However, we observed no difference in the mortality rates of the susceptible “Ngousso” strain when these were exposed to old, used nets and to new, unused nets, indicating that the treated nets had retained some efficacy even after several years of use.

Longevity is the first life trait parameter to be investigated as the lifespan of a mosquito is an essential element in vector control. This parameter defines the age of a mosquito and therefore its capacity to acquire the parasite, allow its development and finally transmit it to a host [50].

A significant difference was observed between the mortality of mosquitoes in the exposed group and that in the control, indicating that exposure to the Olyset bednet negatively affects the life span of vectors. This reduction could be an epidemiological barrier to* Plasmodium* transmission because the vectors must live sufficiently long to ensure parasite transmission [27]. These observations are similar to those made by Tchakounte et al. with the PermaNet 2.0 [21]. Taken together, these results indicate that although the nets are not killing mosquitoes immediately, there is a delayed mortality which could limit the transmission.

*CYP6P9a* and L119F-*GSTe2* were shown to be associated with resistance to insecticide, with *GSTe2* mainly involved in DDT resistance [34] and at a moderate level to permethrin, whereas *CYP6P9a* is highly implicated in resistance to pyrethroid [51]. Both resistance markers are involved in the metabolic detoxification of insecticides and were used in this study to assess a possible association with post-exposure survival. We found that mosquitoes with the* CYP6P9a*-RR genotype survived longer after exposure to the net than their counterparts with the* CYP6P9a*-RS and* CYP6P9a*-SS genotypes, respectively. The same pattern was observed with the L119F-*GSTe2*, but at a limited level. These results suggest that the resistant allele for these markers not only bestows the ability to survive after exposure to the pesticide, as reported previously [51], but could also help mosquitoes to overcome the deleterious effect of the insecticide serveral days after the exposure. Such an increased ability of* GSTe2*-resistant mosquitoes to survive a longer period after exposure was reported previously by Tchakounte et al. [21]. However, our study is the first to show the association between P450-based resistance and post-exposure survival. In this study, we observed that the impact of *CYP6P9a* was stronger than that of *GSTe2* when mosquitoes were exposed to the pyrethroid nets, which is not surprising as *GSTe2* is mainly involved in DDT resistance [34]. These results provide evidence that continued exposure of mosquitoes to bednets will significantly contribute to the selection of resistant alleles and consequently increase malaria transmission. However, rotating insecticides with a different mode of action could help manage this resistance as in the absence of selection resistant mosquitoes are at a disadvantage compared to the susceptible mosquitoes, as previously shown for both markers [52–54]. In fact, P450 monooxygenases and GSTs are known to significantly alter the levels of reactive oxygen species in insects [55]. GSTs protect mosquitoes from oxidative stress, resulting in increased longevity, whereas overproduction of P450 monooxygenases results in increased oxidative stress and thus reduced insect longevity [56]. This shows that in areas of high P450 resistance, rotation of nets could be more successful compared to localities where resistance is mainly driven by GSTs.

## Conclusion

This study has shown that although standard nets have drastically lost their efficacy against resistant field mosquitoes, they still induce a delayed mortality. Nevertheless, the increased longevity of resistant mosquitoes, based on our evaluation of the *CYP6P9a* and *GSTe2* markers, indicates that insecticide resistance could harm the effectiveness of vector control and increase malaria transmission. Therefore, it would be preferable for the NMCP to opt for new-generation nets for future mass distribution campaigns to effectively reduce malaria transmission, as these nets are more effective against pyrethroid-resistant mosquitoes.

## Supplementary Information

**Additional file 1: Table S1.** Different nets collected in Yaoundé. **Table S2**. Different nets collected from households in Elendé.

## Data Availability

Data supporting the conclusions of this article are included within the article. And its additional fles.
