# Supplementary material for: Reduced performance of community bednets against pyrethroid-resistant Anopheles funestus and Anopheles gambiae, major malaria vectors in Cameroon
Source: Parasit Vectors. 2022 Jun 26;15:230. doi: 10.1186/s13071-022-05335-2 (PMC9233849; doi:10.1186/s13071-022-05335-2)
Supplement: Supplementary file 1 — Additional file 1: Table S1. Different nets collected in Yaoundé. Table S2. Different nets collected from households in Elendé. [file 13071_2022_5335_MOESM1_ESM.docx]

**Table S1**. Different nets collected in Yaoundé.

|  | BRANDS | | | | | |  |
| --- | --- | --- | --- | --- | --- | --- | --- |
| **SITES** | **Olyset®Net** | **Super Net** | **PermaNet® 2.0** | **Yorkool®** | **Royal Sentry®** | **Total** |  |
| **ACMS** | - | 05 | - | - | - | 05 |  |
| **Elig-Edzoa** | 01 | - | 03 | 03 | - | 06 |  |
| **Mokolo** | - | - | 03 | 03 | 03 | 09 |  |
| **Central** | 03 | - | - | 03 | 03 | 09 |  |
| **Pharmacies** | - | 10 | - | - | - | 10 |  |
| **Health center** | 02 | - | - | 03 | - | 05 |  |
| **Total**  **(%)** | **06 (13%)** | **15**  **(34%)** | **06**  **(13%)** | **12**  **(27%)** | **06**  **(13%)** | **45** |  |

**Table S2**. Different nets collected from households in Elendé.

| **SITES** |  | BRANDS | | | | |  |
| --- | --- | --- | --- | --- | --- | --- | --- |
| **Households Elende** | **Olyset®Net** | **PermaNet® 2.0** | **Yorkool®** | **Interceptor®** | **PandaNet® 2.0** | **No identified** | **Total** |
| **Total**  **(%)** | **19**  **(63%)** | **05**  **(17%)** | **02**  **(7%)** | **01**  **(3%)** | **01**  **(3%)** | **02 (7%)** | **30** |
